# Efficacy of different treatment protocols for endometritis in *Camelus dromedarius*

**DOI:** 10.3389/fvets.2023.1136823

**Published:** 2023-03-20

**Authors:** Hany Ahmed Zaher, Abdullah F. Al-Fares, Ayman Mesalam

**Affiliations:** ^1^Research and Development Division, Abu Dhabi Agriculture and Food Safety Authority, Abu Dhabi, United Arab Emirates; ^2^Department of Theriogenology, Faculty of Veterinary Medicine, Zagazig University, Zagazig, Egypt

**Keywords:** endometritis, fertility indexes, *Camelus dromedarius*, ceftiofur, oxytetracycline, lotagen

## Abstract

Endometritis is considered a significant cause of infertility problems in dromedary camels. This study aimed to compare the efficacy of different treatment protocols for endometritis in dromedary camels under Abu Dhabi Emirates' conditions. A total of 112 dromedary she-camels with uterine infection were subjected to uterine swabbing for bacterial culture and received one of the following treatments: (i) uterine douching with lotagen every other day for three doses, (ii) single parenteral oxytetracycline injection, (iii) subcutaneous injection with ceftiofur for 5 days, or (vi) combined oxytetracycline-ceftiofur injection. The results showed that *Escherichia coli* was the most isolated bacteria, followed by Streptococcus species. Treatment efficacy was (*P* < 0.05) higher in ceftiofur and oxytetracycline-ceftiofur protocols compared with lotagen and oxytetracycline protocols. The fertility indexes, services per conception and pregnancy rate, were improved in ceftiofur and mixed oxytetracycline plus ceftiofur protocols as the pregnancy rate was (*P* < 0.05) higher in those protocols compared with lotagen and oxytetracycline protocols (71.4 and 67.9% vs. 39.3 and 42.9%, respectively). On the other hand, the number of services per conception was significantly lower in ceftiofur and oxytetracycline-ceftiofur protocols (1.2 for each protocol) than in lotagen and oxytetracycline protocols (1.8 and 1.7, respectively). In conclusion, subcutaneous injection of 1 ml ceftiofur per 50 kg body mass for 5 days can be used as an efficient treatment for uterine infection in female dromedary camels caused by *E. coli* and Streptococcus species for improving their fertility indexes.

## 1. Introduction

Camelids, *Camelus dromedaries*, are the primary resource for livestock products in arid and semiarid areas since they can produce and reproduce under harsh desert conditions (1–3). However, infertility due to uterine infections and high rates of embryo mortality represented a significant complaint in practice (4). Other scholars reported that the foremost reason for reproductive failure in dromedary camels was endometritis (5). Infections of the genital tract are responsible for numerous diseases that contribute to permanent or temporary infertility problems in Camelidae (1).

In camels, due to the relatively short breeding seasonality, any postponement in the treatment of infertility problems triggered the failure of conception and increased the calving intervals, which was linked with economic losses due to the culling of valuable breeding animals (5). To date, most veterinarians used treatments of uterine infections proposed for the bovine or equine species for the treatment of uterine diseases in female camels (6). Therefore, isolating bacteria from the reproductive organs to diagnose and intervene in infertility conditions was considered a milestone for managing poor reproductive performance in Camelidae. Because of the broad diversity of bacteria implicated in uterine infections, performing antibiotic sensitivity tests was indispensable for the successful treatment of endometritis (7). But, as most causative organisms were omnipresent, the culture results can be misleading especially if the clinical findings were not considered (8).

Clinical endometritis is the most common clinical finding detected in female dromedary camels with infertility problems (9). Surprisingly, leaving uterine infections without treatment can lead to severe uterine damage, irreversible changes, and incurable complications such as salpingitis and total fertility loss. Following parturition, bacterial contamination of the uterus frequently continues; uterine infections developed and cause infertility (10). The most common conventional method used to overcome the adverse effect of uterine infection on camel fertility is the application of intrauterine antibiotics (11). However, the negative interactions between the antibiotic and the uterine environment made its efficacy questionable (12). Subsequently, the parenteral application of several broad-spectrum antibiotics as an alternative approach has been repeatedly established over the last decade (11).

We hypothesized that determining the most common isolated bacteria from the uterus of she-camels affected with endometritis could help in selecting the most appropriate antibiotic. And that can improve the reproductive efficiency of camels *via* the effective treatment of endometritis and enhance the fertility indexes in terms of services per conception and pregnancy rate. Thus, the current study aimed to determine the most causative bacteria for endometritis in camels and evaluate the efficiency of four regimes for treating endometritis in female dromedary camels. Also, to assess the fertility indexes of the mated females following treatment with different treatment regimes.

## 2. Materials and methods

### 2.1. Ethical approval

The protocols and procedures were approved by the Research and Development Division, Abu Dhabi Agriculture and Food Safety Authority, Abu Dhabi, United Arab Emirates.

### 2.2. Animal management

The study was conducted on 112 female dromedary camels with a history of conception failure during the breeding season (2020–2021) at the artificial insemination and embryo transfer section, Abu Dhabi Agriculture and Food Safety Authority, Abu Dhabi, United Arab Emirates. Camels were housed in 8 × 8 m open yards, shaded with east-west oriented slopped shades 5–8 m high and made by reed mats. Animals were fed alfalfa and Rhodes grass (7 kg/head twice daily) to meet their nutritional requirement, and they got clean water *ad libitum* (13).

### 2.3. Gynecological examination

The vaginal examination was performed by speculum for inspection of the vaginal mucus membrane, followed by the trans-rectal ultrasound examination using a 5 MHz linear-array transducer (SSD ProSound 2, Model UST-5820-5C, Aloka^®^, Tokyo, Japan) to assess the conditions of the reproductive organs. The presence of any abnormal uterine content, which increased the uterine size and the uterine wall thickness, was recorded.

### 2.4. Bacterial culture

Swabs were collected from the uterus using a double-guarded culture swab (Equivet^®^, Langeskov, Denmark) to determine the most common contributing bacteria as previously described (1). Briefly, the protecting outer sheath and cap were penetrated and the swab was passed through the cervix toward the base of uterine horns by rectal manipulations. After gentle rotation for 30 s against the endometrium, the swab was retracted to the protecting guards. Obtained swabs were preserved in a transporting medium (Amies clear medium) and transported to the laboratory within 3 h for bacterial culture and isolation. Swabs were gently squeezed against the tube wall, inoculated onto MacConkey agar and sheep blood agar, and incubated invertedly for 24–48 h at 37°C. The grown colonies were visually inspected and subjected to MALDI-TOF MS system (VITEK^®^ MS, bioMérieux, France) for identification.

### 2.5. Experimental design and animal treatment

Based on the gynecological examination, animals with serous or no vaginal discharge, normal uterine lumen, and uterine thickness from 6 to 9 mm have been associated with endometritis, which indicated a proven history of conception failure, were divided randomly into four groups, twenty-eight animals per group, and received one of the following therapeutic protocols.

#### 2.5.1. Uterine douching with Lotagen

Three uterine douches were performed as previously reported (8) using 120 ml lotagen (Bioveta, Czech) using disposable uterine catheters (Bovivet; Kruuse, Denmark). The treatment was repeated every other day.

#### 2.5.2. Oxytetracycline injection

Animals received a single intramuscular injection with 20 mg oxytetracycline per kg live body mass (Terramycin^®^ LA; Zoetis, Spain).

#### 2.5.3. Ceftiofur injection

Animals received subcutaneous injection with 1 ml ceftiofur per 50 kg body mass (Ceftionel-50; Interchemie, Netherlands) for 5 days.

#### 2.5.4. Ceftiofur-oxytetracycline injection

Animals received oxytetracycline-ceftiofur as the above-recommended doses for each antibiotic.

### 2.6. Assessment of recovery and pregnancy rates

All the animals were followed up and investigated for normal uterine thickness (5–6 mm) by ultrasound device for complete recovery 14 days from the treatment. Sexually receptive females were mated only once, and those returned to estrus were rebred again. Service per conception (S/C) was calculated by the number of estrus periods during which mating took place. Pregnancy diagnosis and confirmation were made by ultrasonography (Supplementary Figure 1), and the pregnancy rate was calculated by dividing the number of camels diagnosed as pregnant 28 days after mating by the total number of camels mated.

### 2.7. Statistical analysis

Data were analyzed using a statistical software program (SPSS, version 22, IBM Corp., Armonk, NY), and the differences between groups were analyzed using either Student's *t*-test or one-way analysis of variance (ANOVA). Duncan's was applied to determine the significance level between groups. The *p* < 0.05 were considered statistically significant.

## 3. Results

### 3.1. Bacteriological examination and ultrasonography evaluation

The bacteriological examination of genital discharge revealed that *Escherichia coli* was the most common isolated bacteria (50%), followed by Streptococcus species (37.5%) (Table 1). At the same time, Bacteroides and Moraxella species were isolated in only 25 and 12.5%, respectively. Ultrasonographic investigation revealed an increase in uterine thickness in diseased animals which was (*P* < 0.05) reduced following different treatment regimes (Table 2; Supplementary Figure 2).

**Table 1:** Results of microbiological examination performed on genital swabs collected from dromedary camels with endometritis and a history of conception failure.

	***Escherichia coli***	**Streptococcus species**	**Bacteroides**	**Moraxella species**	**Total**
*Escherichia coli*	28/112 (25%)	10/112 (8.9%)	13/112 (11.6%)	5/112 (4.5%)	56/112 (50%)
Streptococcus species	10/112 (8.9%)	32/112 (28.6%)	–	–	42/112 (37.5%)
Bacteroides	13/112 (11.6%)	–	15/112 (13.4%)	–	28/112 (25%)
Moraxella species	5/112 (4.5%)	–	–	9/112 (8.0%)	14/112 (12.5%)

**Table 2:** Results of uterine thickness (cm) before and after treatment of endometritis in dromedary camels using different treatment regimes.

	**Before treatment**	**After treatment**
Lotagen	0.77 ± 0.08	0.62 ± 0.07^*^
Oxytetracycline	0.77 ± 0.08	0.59 ± 0.05^*^
Ceftiofur	0.77 ± 0.08	0.57 ± 0.03^*^
Combined oxytetracycline-ceftiofur	0.77 ± 0.08	0.55 ± 0.04^*^

Values with an asterisk in the same row are significantly different (*P* < 0.05).

### 3.2. Recovery rate

**Figure 1:**
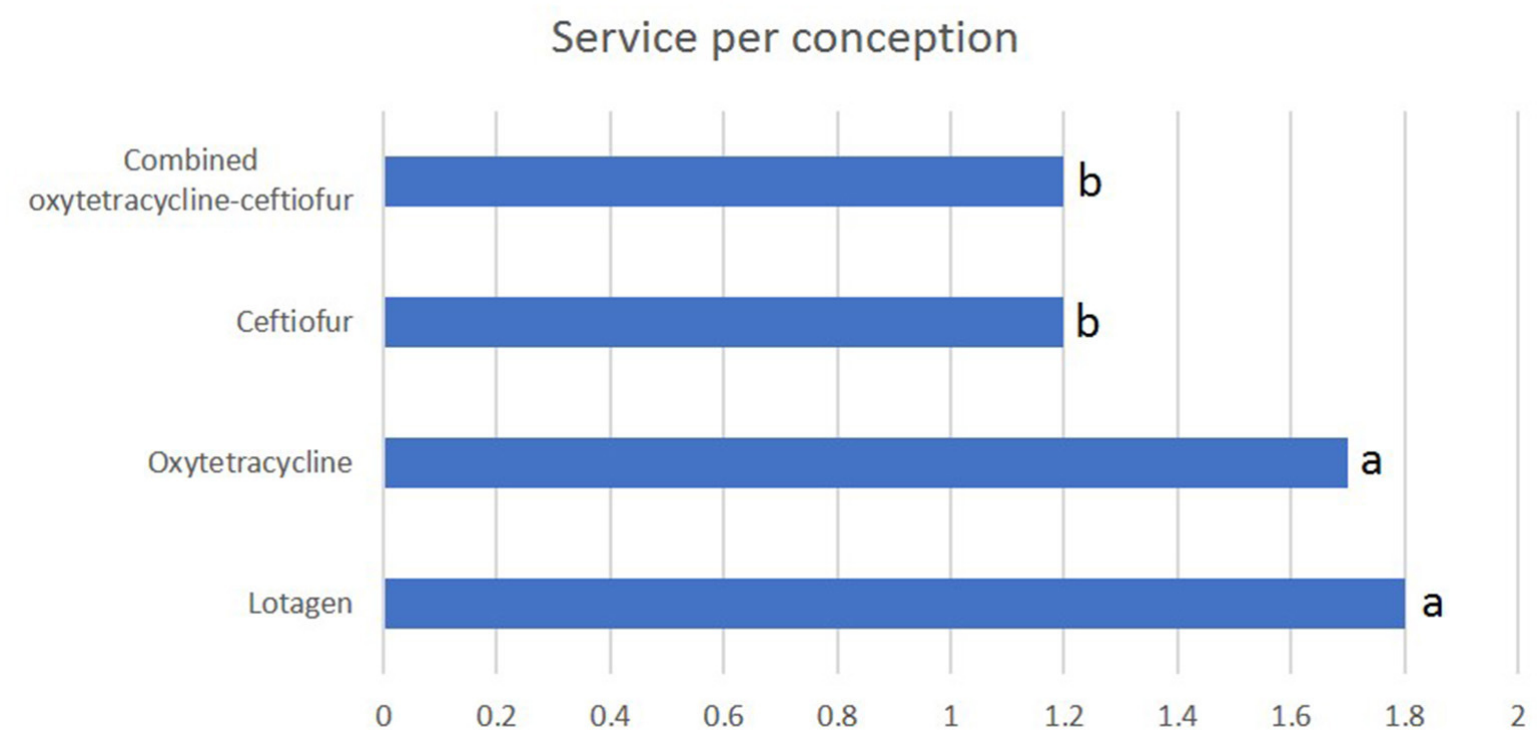
Recovery rate in different protocols used for the treatment of endometritis in dromedary camels. The main criterion for assessment of recovery was returning to normal uterine thickness (5–6 mm) as measured by ultrasound device 14 days post-treatment. Columns with different letters indicate a significant difference (*P* < 0.05).

The recovery rate in ceftiofur and oxytetracycline-ceftiofur protocols were (*P* < 0.05) high (75% for each protocol) compared to lotagen and oxytetracycline protocols (50% for each protocol) (Figure 1). Nevertheless, there was no statistically significant difference (*P* > 0.05) observed between ceftiofur and oxytetracycline-ceftiofur protocols or between lotagen and oxytetracycline protocols (Figure 1).

### 3.3. Pregnancy rate

**Figure 2:**
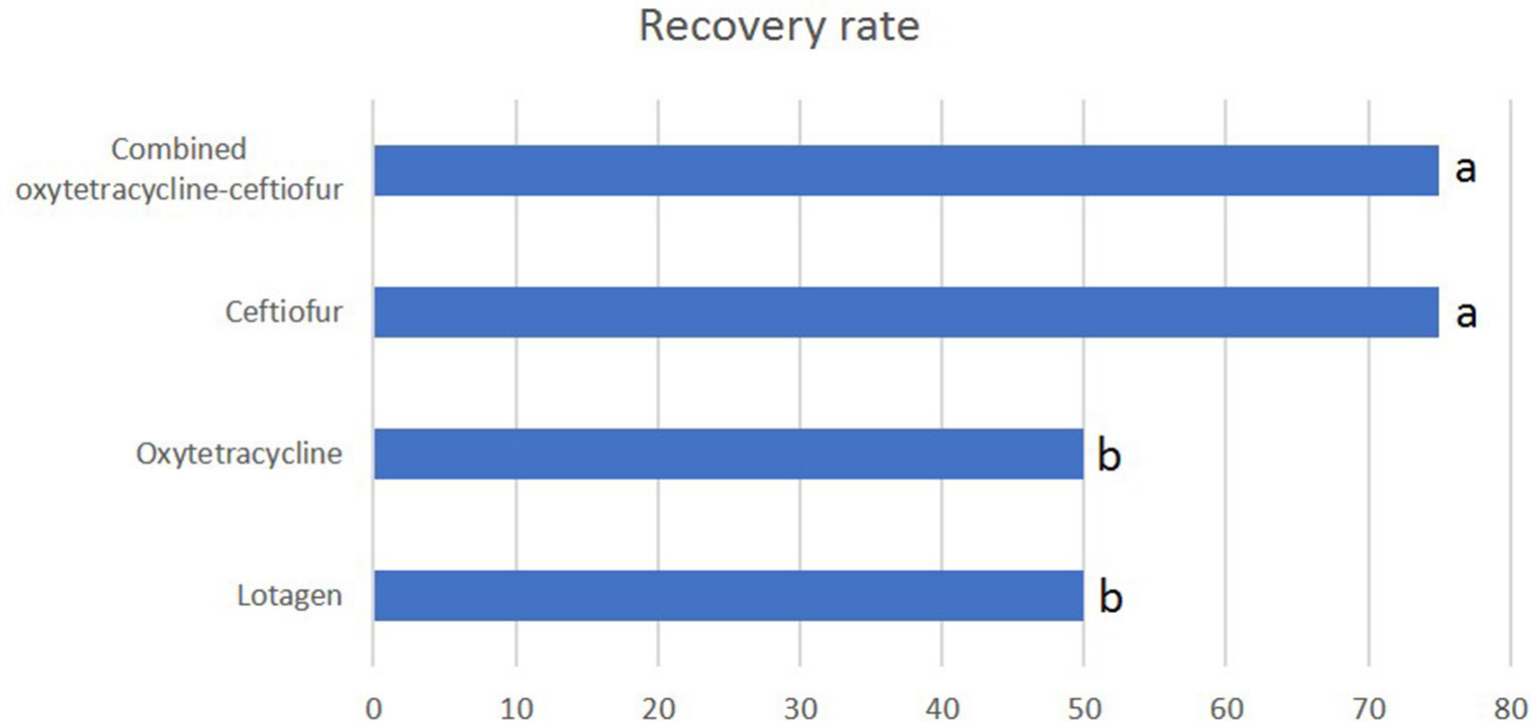
The pregnancy rate in different protocols used for the treatment of endometritis in dromedary camels. The pregnancy rate was calculated by dividing the number of camels diagnosed as pregnant 28 days after mating by the total number of camels mated. Columns with different letters indicate a significant difference (*P* < 0.05).

The pregnancy rate in the ceftiofur protocol was (*P* < 0.05) high compared with lotagen and oxytetracycline protocols (71.4 vs. 39.3 and 42.9%, respectively; Figure 2). Similarly, the pregnancy rate in the oxytetracycline-ceftiofur protocol was (*P* < 0.05) high compared with lotagen and oxytetracycline protocols (67.9 vs. 39.3 and 42.9%, respectively; Figure 2). No differences (*P* > 0.05) between ceftiofur and oxytetracycline-ceftiofur protocols or between lotagen and oxytetracycline protocols were observed (Figure 2).

### 3.4. Service per conception

**Figure 3:**
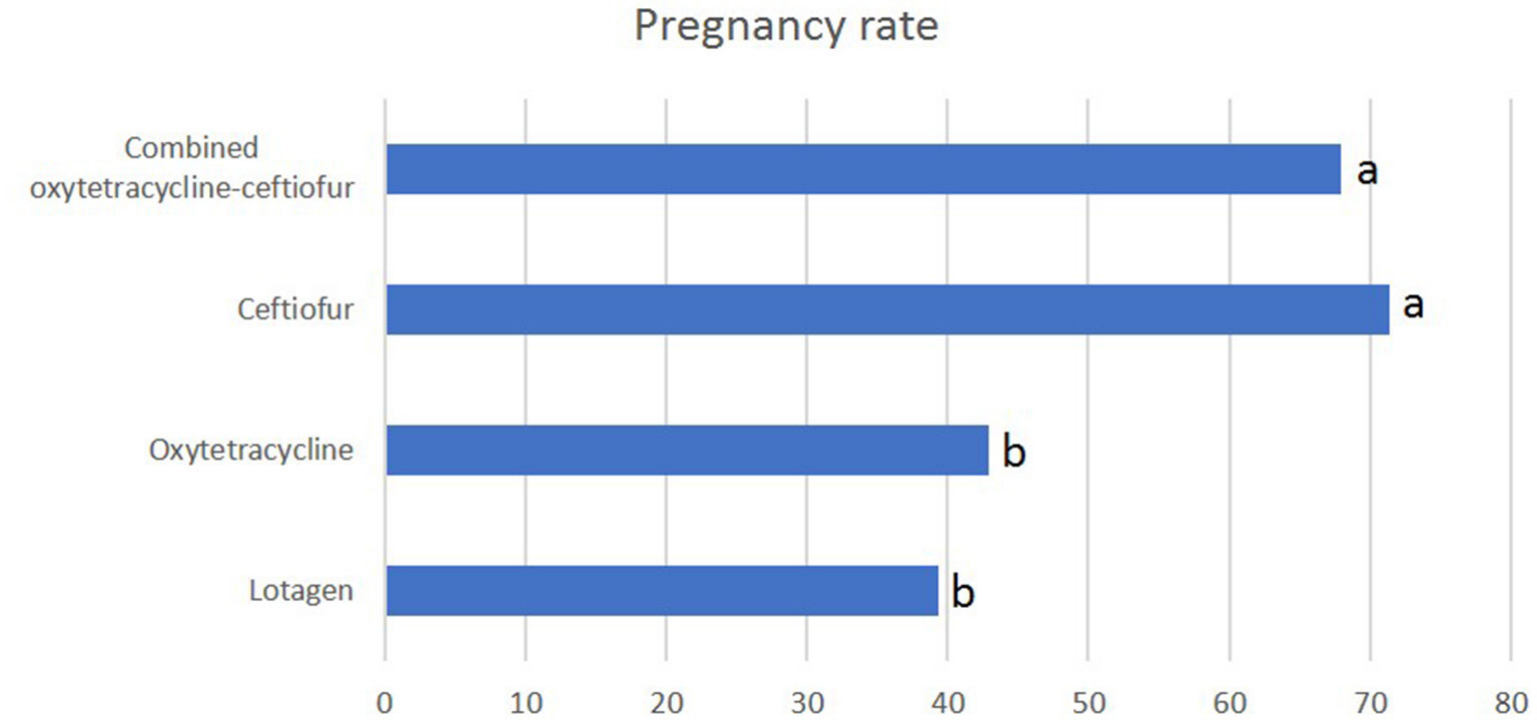
Service per conception in different protocols used for the treatment of endometritis in dromedary camels. Service per conception was calculated by the number of estrus periods during which mating took place. Columns with different letters indicate a significant difference (*P* < 0.05).

The number of services per conception in ceftiofur and oxytetracycline-ceftiofur protocols was significantly (*P* < 0.05) lower (1.2 for each protocol) than lotagen and oxytetracycline protocols (1.8 and 1.7, respectively) (Figure 3). No (*P* > 0.05) differences between ceftiofur and oxytetracycline-ceftiofur protocols or between lotagen and oxytetracycline protocols were observed (Figure 3).

## 4. Discussion

Over the past decades, Camelidae has been recognized for their lower reproductive efficiency despite their cherished involvement in production resources (meat, milk, and hair fiber production) (14, 15). Camel reproductive diseases, uterine infections, repeat breeding, early embryonic death, and fetal loss were mentioned to be the significant problems that caused infertility in she-camels (16). The major contributing factors for uterine infections are overbreeding, infection of the genitalia during parturition, postpartum complications, and unhygienic gynecological manipulation (17). Yet, camels' reproduction problems are not as widely investigated as in other animal species. In this study, we determined the most isolated bacteria accused of causing endometritis and evaluated the efficiency of four different endometritis-treatment regimes in dromedary camels.

In the current study, *Escherichia coli* was the most commonly isolated bacteria from uterine swabs, followed by Streptococcus species. These findings agree with previous studies reported that *Escherichia coli, Streptococcus zooepidemicus*, and β*-haemolytic Streptococci* were the most isolated bacteria from she-camels with endometritis (18–20). Additionally, Tibary et al. reported that the primary pathological organisms incriminated in metritis and endometritis in camelids were *Escherichia coli* and *Streptococcus equi* subspecies *zooepidemicus* (1).

It is broadly recognized that in case of uterine infections, the ultrasonography examination may reveal an increase in the uterine wall thickness, accumulation of fluid in the uterine lumen, or both (1). Moreover, endometritis may be associated with echogenic fluid accumulated within the uterine lumen in mares (21) or echogenic line consistently appearing in the uterine longitudinal section in dairy cows (22). Concerning the present findings, the ultrasonographic results showed an increase in uterine thickness in the case of endometritis which may be attributed to the thickening and hypertrophy of the blood vessels and infiltration of fibroblasts and inflammatory cells in subepithelial tissue. This was comparable with previous reports showed that buffaloes' genitalia exhibited thickening of uterine walls and the presence of varying degrees of exudate in uterine infections samples (23).

It has been documented that metritis and endometritis can be effectively treated using the either parenteral or intrauterine application of antibiotics or both (7, 24, 25). Remarkably, in dairy cows, puerperal infections have been extensively treated with parenteral administration of antibiotics (26). It has been reported that ceftiofur can be effectively used against most gram-positive and -negative pathogens (27). In Europe and the United States, ceftiofur has been officially approved for treating cows with different pathological conditions such as postpartum metritis, respiratory disease, and interdigital necrobacillosis (28). In addition, ceftiofur, either intramuscular or subcutaneous injections, was used for treating metritis in dairy cows (24). The results from the current study reported that camels treated with ceftiofur or oxytetracycline-ceftiofur showed higher recovery rates than those treated with lotagen or oxytetracycline. This confirms the previous studies which reported the parenteral treatment of cattle with toxic puerperal metritis using ceftiofur is a practical alternative to using a combination of local and parenteral therapies (29).

The reproductive performance is reduced by uterine infection, as services/conception, days open, and calving to the first service interval were reported to be higher in cows with uterine infection (30, 31). Our results showed that the fertility indexes, services per conception and pregnancy rate, were better for female camels of the ceftiofur and mixed oxytetracycline plus ceftiofur groups than for those of the other treated groups, which is supported by a previous study that reported good effectiveness of ceftiofur in puerperal metritis treatment in dairy cows (29). Several publications have demonstrated that pregnancy rates after treatment of endometritis vary from 30 to 60% (20, 32), and the conception rate obtained after treatment with lotagen was 49.3% (33). The possible reason for the lower efficacy of the intrauterine infusion of oxytetracycline could be the lower sensitivity of the causative bacteria to the treatment (34).

## 5. Conclusions

It could be concluded that ceftiofur, a third-generation broad-spectrum cephalosporin antibiotic, was the best protocol used in she-camels with uterine infection and achieved a higher pregnancy rate following recovery. Otherwise, the other evaluated regimes, oxytetracycline injection and uterine douching with lotagen were less efficient in treating females with uterine infections. This study provides new insight into affording relevant guidelines and instructions for those protocols for veterinarians and camel breeders and generalizes the use of ceftiofur for treating infertility in she-camels.

## Data availability statement

The raw data supporting the conclusions of this article will be made available by the authors, without undue reservation.

## Ethics statement

The animal study was reviewed and approved by Research and Development Division, Abu Dhabi Agriculture and Food Safety Authority, Abu Dhabi, United Arab Emirates.

## Author contributions

HZ conceived, designed, and performed the experiments. AA-F analyzed the data. AM wrote the paper. HZ and AM revised and edited the manuscript. All authors have read and agreed to the published version of the manuscript.
