# Supplementary material for: Efficacy of different treatment protocols for endometritis in Camelus dromedarius
Source: Front Vet Sci. 2023 Mar 20;10:1136823. doi: 10.3389/fvets.2023.1136823 (PMC10069631; doi:10.3389/fvets.2023.1136823)
Supplement: Supplementary file 1 [file Data_Sheet_1.docx]

Supplementary Material

Efficacy of Different Treatment Protocols for Endometritis in *Camelus dromedaries*

Hany Ahmed Zaher, Abdullah F Al-Fares, Ayman Mesalam^*^

*** Correspondence:** Ayman Mesalam: aymanmesalam@gmail.com


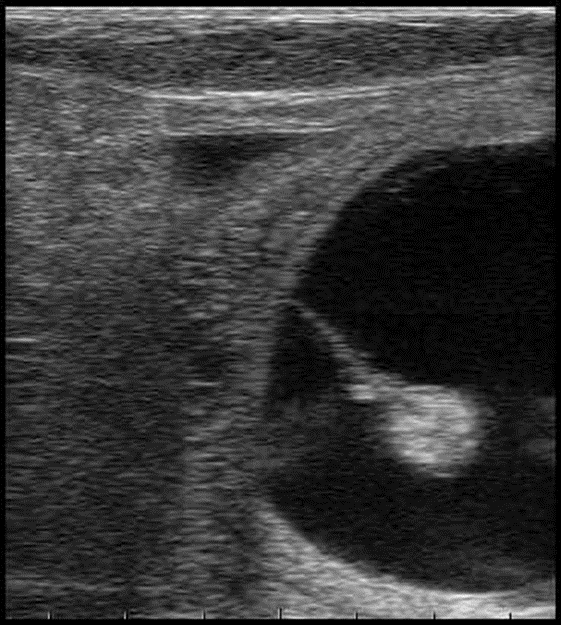

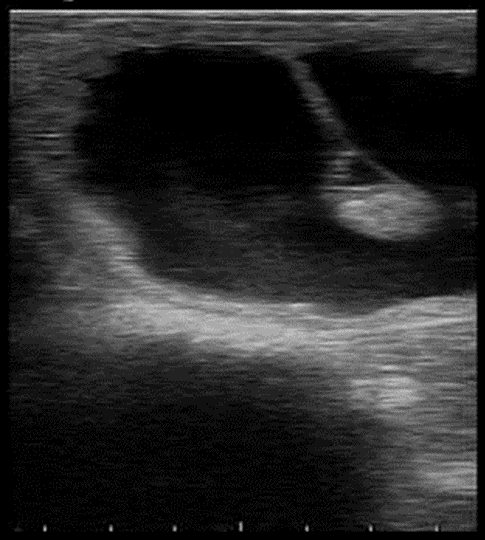


**A**

**B**

**Supplementary Figure 1.** Ultrasound imaging of gravid uterus of camel treated with different protocols used for treatment of endometritis in dromedary camels. (A) Gravid uterus with 32-days pregnancy. (B) Gravid uterus with 34-days pregnancy. Pregnancy diagnosis was performed using trans-rectal ultrasound examination using a 5 MHz linear-array transducer (SSD ProSound 2, Model UST-5820-5C, Aloka®, Tokyo, Japan).


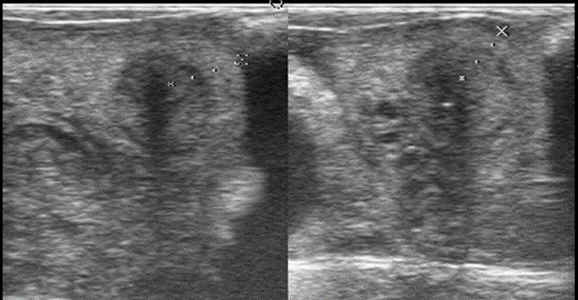

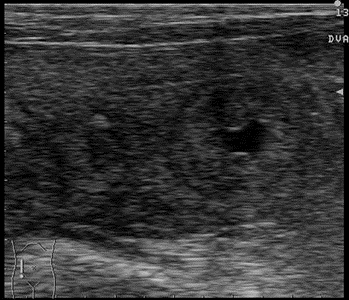


**A**

**B**

**Supplementary Figure 2.** Ultrasound imaging of the uterus of dromedary camels affected with endometritis. (A) Cross section of the uterus of dromedary camels affected with endometritis showing accumulation of echogenic fluid within the uterine lumen during the follicular phase of the cycle. (B) Cross section of the uterus of dromedary camels affected with endometritis showing increased endometrial thickness of both right and left uterine horns during the luteal phase of the cycle. Ultrasound examination was performed using trans-rectal 5 MHz linear-array transducer (SSD ProSound 2, Model UST-5820-5C, Aloka®, Tokyo, Japan).
